# Thymic Output and CD4 T-Cell Reconstitution in HIV-Infected Children on Early and Interrupted Antiretroviral Treatment: Evidence from the Children with HIV Early Antiretroviral Therapy Trial

**DOI:** 10.3389/fimmu.2017.01162

**Published:** 2017-09-20

**Authors:** Joanna Lewis, Helen Payne, A. Sarah Walker, Kennedy Otwombe, Diana M. Gibb, Abdel G. Babiker, Ravindre Panchia, Mark F. Cotton, Avy Violari, Nigel Klein, Robin E. Callard

**Affiliations:** ^1^CoMPLEX, University College London, London, United Kingdom; ^2^NIHR Health Protection Research Unit in Modelling Methodology, Department of Infectious Disease Epidemiology, Imperial College London, London, United Kingdom; ^3^Institute of Child Health, University College London, London, United Kingdom; ^4^MRC Clinical Trials Unit, University College London, London, United Kingdom; ^5^Perinatal HIV Research Unit, Faculty of Health Sciences, University of the Witwatersrand, Johannesburg, South Africa; ^6^Children’s Infectious Diseases Clinical Research Unit, Department of Paediatrics and Child Health, Stellenbosch University, Stellenbosch, South Africa

**Keywords:** HIV, children, antiretroviral therapy, planned treatment interruption, CD4 T cells, CD4 count, thymus

## Abstract

**Objectives:**

Early treatment of HIV-infected children and adults is important for optimal immune reconstitution. Infants’ immune systems are more plastic and dynamic than older children’s or adults’, and deserve particular attention. This study aimed to understand the response of the HIV-infected infant immune system to early antiretroviral therapy (ART) and planned ART interruption and restart.

**Methods:**

Data from HIV-infected children enrolled the CHER trial, starting ART aged between 6 and 12 weeks, were used to explore the effect of ART on immune reconstitution. We used linear and non-linear regression and mixed-effects models to describe children’s CD4 trajectories and to identify predictors of CD4 count during early and interrupted ART.

**Results:**

Early treatment arrested the decline in CD4 count but did not fully restore it to the levels observed in HIV-uninfected children. Treatment interruption at 40 or 96 weeks resulted in a rapid decline in CD4 T-cells, which on retreatment returned to levels observed before interruption. Naïve CD4 T-cell count was an important determinant of overall CD4 levels. A strong correlation was observed between thymic output and the stable CD4 count both before and after treatment interruption.

**Conclusion:**

Early identification and treatment of HIV-infected infants is important to stabilize CD4 counts at the highest levels possible. Once stabilized, children’s CD4 counts appear resilient, with good potential for recovery following treatment interruption. The naïve T-cell pool and thymic production of naive cells are key determinants of children’s CD4 levels.

## Introduction

In 2014, 2.6 million children were living with HIV worldwide, and under-15s experienced 220,000 new infections, and 150,000 AIDS-related deaths (1). Despite efforts to extend programs to prevent vertical transmission, only 77% of HIV-infected pregnant women in 21 priority countries received antiretroviral therapy (ART) (2). For some years to come, the optimal management of perinatally acquired HIV infection is likely to remain an important issue.

The Children with HIV Early Antiretroviral Therapy (CHER) trial (3) provided compelling evidence for early ART initiation in perinatally infected infants. Infants enrolled either received immediate, time-limited ART followed by planned interruption, or deferred treatment until meeting predefined clinical and immunological criteria (4). Those randomized to deferred treatment experienced rapid disease progression and increased mortality compared to children treated immediately. In response, WHO guidelines changed to recommend universal treatment for all children under 1 year of age (4, 5).

Several studies have described CD4 dynamics in older children and adults starting, interrupting, and restarting ART. Higher CD4 counts are achieved in the long term if children start therapy at younger ages or with higher pre-ART CD4 counts (6, 7). Children restarting ART after planned treatment interruption can recover CD4 levels similar to those on continuous ART (8). In adults, the effects of interruption are very different: CD4 counts on restarted therapy following relatively long interruptions remain lower than if on continuous treatment (9, 10). The reasons for the differences between children and adults are unclear, but one contributing factor may be the much higher thymic export of naïve T-cells in children than in adults (11). Thymic activity is decreased in children with HIV infection but restored to normal levels in response to treatment (12) and may play a role in T-cell reconstitution.

It is also known that there is significant immunological variation between individuals, which is influenced by age, sex, genetics, antigenic exposure, and other environmental factors (13). CD4 counts in healthy children vary with age, and there is also significant variation between different children of the same age (14, 15). When HIV-infected children start ART, they show a range of CD4 responses that were associated with age, pre-ART CD4 levels and virological response to treatment (7). In any analysis of CD4 response to ART, it is important to consider interindividual differences and their potential causes.

We investigated patterns of T-cell homeostasis and reconstitution in infants interrupting ART during the CHER trial. We show that early ART stabilized CD4 levels, but did not increase counts to those seen in uninfected children. Following treatment interruption, ART restored CD4 counts to at least pre-interruption levels. Thymic output was a key determinant of both pre- and post-ART CD4 counts. This information contributes to our understanding of T-cell homeostasis in healthy and HIV-infected children.

## Materials and Methods

### The CHER Trial

**Figure 1:**
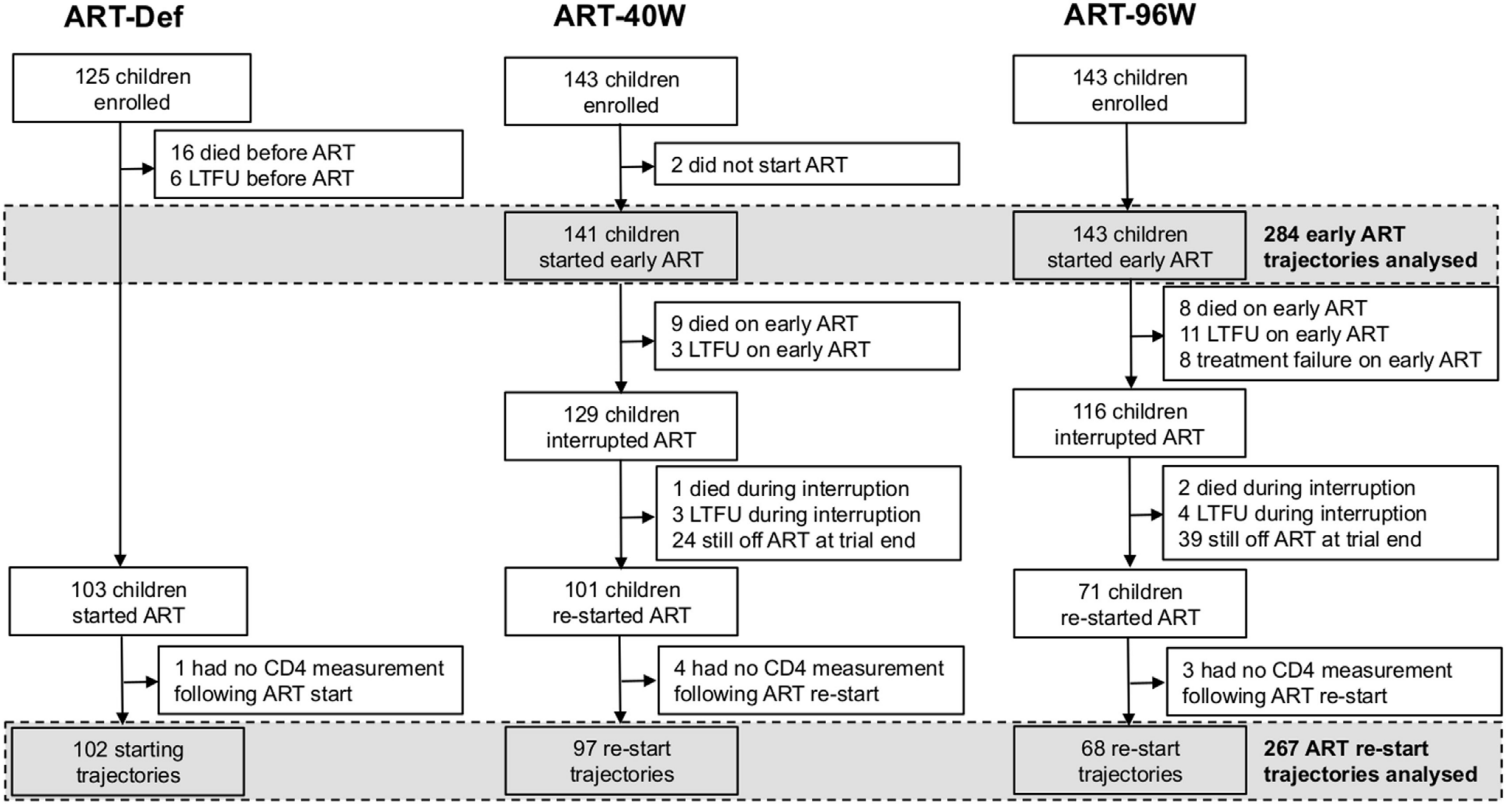
Enrollment, dropout, and antiretroviral therapy (ART) of children in the children with HIV early antiretroviral therapy trial. LTFU, lost to follow-up.

Children with HIV early antiretroviral therapy was a two-center randomized clinical trial comparing three treatment strategies in infants with perinatally acquired HIV-1 infection diagnosed between 6 and 12 weeks of age. Infants were recruited through PMTCT programs at the Perinatal HIV Research Unit (PHRU) in Soweto, Johannesburg and the Children’s Infectious Diseases Clinical Research Unit (KIDCRU) in Tygerburg, Cape Town, South Africa. Research ethics committees in South Africa and the USA approved the trial. Parents or guardians gave written consent for screening and enrollment. Children with CD4% ≥25% at screening were eligible for randomization to one of three ART regimens: deferred treatment (ART-Def), and either 40 weeks (ART-40W) or 96 weeks (ART-96W) of early treatment starting between 6 and 12 weeks. The ART-40W and ART-96W groups then interrupted treatment, except in a few cases of treatment failure where a trial endpoint was met (Figure 1). Children were monitored during treatment interruption, and continuous therapy was re-initiated according to predefined clinical, immunological, or virological criteria (3). Children were seen every 4 weeks from enrollment until week 24, eight-weekly to week 48 and 12-weekly thereafter. In this analysis, we consider the CD4 counts collected at clinic visits. The protocol and primary results are reported fully elsewhere (3).

To be included in our analysis, children had to have CD4 measurements made while on early and/or continuous ART. Children who did not start early ART were, therefore, excluded from our analysis of early ART trajectories. The analysis of continuous ART trajectories excluded children who did not interrupt ART, who did not (re-)start continuous ART during the follow-up period, or who (re-)started but had no continuous-ART CD4 measurements available (Figure 1).

In a substudy of CHER, naive and memory T-cell levels were measured retrospectively by flow cytometry using cryopreserved peripheral blood mononuclear cells (PBMCs). We analyzed at most one timepoint per participant during early therapy (chosen at random if more than one early-therapy timepoint per child was available). We also considered the first available timepoint on continuous therapy, included only if measured at <20 weeks after restarting ART, and the last available timepoint on continuous therapy, included only if measured >100 weeks after restarting ART.

The original CHER study received ethical approval from the Health Research Ethics Committee of the University of Stellenbosch with reference number M04/07/033A and from the University of the Witwatersrand Human Research Ethics Committee under ethics reference number 040703. The substudy providing the more detailed immunological data was also approved by ethics committees at Stellenbosch University (reference number M12/01/005) and the University of the Witwatersrand (with the same reference: 040703).

### Age-Correction for CD4 Counts

Total naïve and memory CD4 counts vary with age in healthy children, changing particularly rapidly during infancy (14). To compare CD4s in children of different ages and in the same child at different times, *z*-scores based on reference data from HIV-uninfected children born to HIV-infected mothers in Europe were used (15, 16). (Functions for *z*-scores based on healthy South African children are not currently available.) A *z*-score of 0 corresponds to the median CD4 count for a healthy child of the same age, a positive value indicates a count above the median and a negative value a count below the median. Approximately 95% of healthy children have CD4 *z*-scores between −2 and 2.

Naïve and memory cell counts were expressed as the natural logarithm of the ratio of the count to that expected in a healthy child of the same age (14). A value less than 0, therefore, represents a count below average and a value above 0 a level above average for a healthy child:

$$\text{Naive CD4-for-age}=\frac{\text{Naive CD4 count}}{\text{Naive CD4 count expected in a healthy child of the same age}},$$

$$\text{Memory CD4-for-age}=\frac{\text{Memory CD4 count}}{\text{Memory CD4 count expected in a healthy child of the same age}}.$$

It is important to note that neither of the reference datasets used for age-adjusted CD4 counts were collected from Black African children living in Africa although the reference data for CD4 *z*-scores included a number of Black African children living in Europe. Ethnic and/or environmental differences may affect total naïve and memory CD4 counts (17, 18). To compare reference values between the European children used to calculate *z*-scores and Black African children living in Africa, immunophenotypic data were obtained from the Child Wellness Clinic (CWC) for children from birth up to 12 years of age, established at a community health clinic in Wesbank, a suburb in the Eastern subdistrict of Cape Town. The primary aim of the CWC was to acquire HIV-uninfected blood specimens comparable to CHER participants in terms of intercurrent infection, nutrition, vaccination, and socio-economic status to establish hematological and immunological reference ranges (19) and measure thymic output in healthy children. The *z*-scores calculated for this population as described above were comparable to the European *z*-scores and were also compared with HIV-infected children (see Results).

### Characterizing CD4 Trajectories

We previously characterized CD4-for-age trajectories in HIV-infected older children on ART by a combination of non-linear mixed-effects modeling and linear regression (7). Here, we used the same process to classify trajectories in these younger children and infants from the CHER trial restarting ART following interruption.

**Figure 2:**
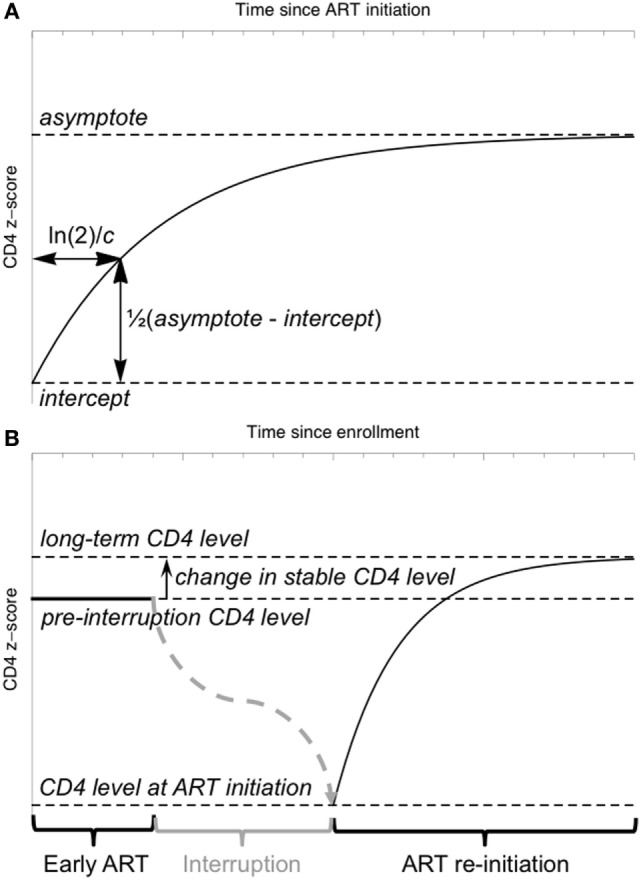
Schematic illustration of the model for CD4 *z*-score trajectories. **(A)** The “asymptotic” model of CD4 *z*-score recovery following antiretroviral therapy (ART) initiation or restart. **(B)** A model of CD4 *z*-score in a child interrupting and restarting ART, including an early stable CD4 *z*-score, a fall in *z*-score during interruption (not modeled) and an asymptotic recovery on ART restart.

Each child’s CD4 *z*-scores were fitted to a curve of the form:

(1) CD4z-score=int−(asy−int)×exp(−ct),

where *t* represents time since ART initiation (Figure 2A). This “asymptotic” pattern of recovery assumes an initially low CD4 *z*-score (the intercept, int) which increases quickly at first, slowing down with time to approach a stable long-term level (the asymptote, asy). The parameter *c* characterizes the rate of this recovery. The aim of this initial model was to separate children with a clear asymptotic pattern of recovery from those with qualitatively different recovery profiles. If the CD4 *z*-score trajectories did not fit the asymptotic pattern (model non-convergence), linear regression was used to identify children in whom there was evidence that CD4 *z*-score was increasing or decreasing (*p* < 0.05 for the null hypothesis of a slope not significantly different from 0), and children in whom there was no evidence of any change (*p* > 0.05).

Preliminary investigations suggested that the asymptotic model was not appropriate for most children during early therapy. CD4 *z*-score trajectories during early therapy were, therefore, classified using only linear regression, as having increasing or decreasing CD4 *z*-score (*p* < 0.05) or no evidence of any trend (*p* > 0.05).

### Mixed-Effects Modeling of Longitudinal CD4 Data

To investigate the relationship between CD4 *z*-scores on early therapy, at ART restart and in the long term, a non-linear mixed-effects model incorporating the asymptotic response function (Eq. 1) was used. CD4 *z*-score on early therapy is assumed constant, as observed in the majority of children. After ART interruption, the CD4 *z*-score falls according to a trajectory not specified in the model, and data collected during interruption was, therefore, excluded from the model fitting. The model included four parameters: the stable *z*-score level while on early ART, the *z*-score when ART was restarted following interruption, the difference between the stable level on early therapy and on long-term restarted therapy, and the time constant characterizing the speed of the asymptotic increase (Figure 2B). This parameterization allows formal statistical testing of the hypothesis that the difference between stable CD4 *z*-scores on early and long-term therapy is not significantly different from 0.

In mixed-effects models, parameter values are inferred for the population on average (fixed effects) and can also be allowed to vary between individual children (random effects). In the model used here, the four parameters were all allowed to vary between children, with non-zero covariance between parameters (i.e., correlation between parameters was allowed). An additional fixed effect was introduced when ART was restarted after treatment interruption to allow a systematic difference between the first CD4 *z*-score observed after therapy restarted and the intercept of the asymptotic curve, as we found that this significantly improved the fit to the data (objective function value (OFV), −2 × log-likelihood, reduced by 17 U). This model was compared to one where the steady-state CD4 *z*-score on initial treatment changed continuously with time since enrollment (rather than undergoing a “step” during interruption) but although the objective function decreased by 8 U, the rate of change was estimated as (−1.47 ± 0.87) × 10^−4^ *z*-score units week^−1^ (value, SE) and there was, thus, no significant evidence of a slope significantly different from 0.

Mixed-effects models also allow predictive effects of child-specific covariates on the model parameters to be investigated. We investigated the effects of study site, sex, weight at birth, age and weight at enrollment (original ART initiation), CDC disease stage at enrollment and duration of early therapy (trial randomization) on all four parameters of the model, and the effects of reason for restarting ART, age at restarting and interruption length on CD4 *z*-score at ART re-initiation, CD4 *z*-score in the long term, and rate of CD4 recovery. We did not use CD4 *z*-score at enrollment as an additional factor, because it was stable and, therefore, its influence on long-term CD4 *z*-scores was already included through the random effects. We used forward selection for building the covariate models, comparing the change in OFV to a χ^2^ distribution and including new models with *p*-values for the new covariate less than 0.1. This was followed by a backwards-elimination phase with a threshold *p*-value of 0.05. (We report *p*-values for the null hypothesis of no significant difference in the fit in this backward-elimination process.) Finally, we checked the effect on OFV of adding further covariates to our final model.

The mixed-effects framework we used for analysis has several advantages. First, it provides a statistically rigorous approach for analyzing longitudinal data, allowing the full power of the whole dataset to inform the results. More traditional methods are usually limited to simpler analyses on a subset of the data: for example, comparing pairs of timepoints. Second, mixed-effects models allow very explicitly for interindividual variability. Immunological variation between individuals is significant and is of particular interest in this analysis of between-child differences in CD4 response and their possible causes. Mixed-effects modeling provides a tool to investigate predictive effects of measured covariates on the CD4 response. Finally, non-linear mixed-effects models can account for complex trial protocols like CHER’s because they are very flexible in the form of the CD4 response that can be proposed.

### Thymic Output

Thymic output was estimated by combining measurements of T cell receptor excision circles (TRECs) from purified naïve CD4 T-cells with Ki67 labeling to calculate the rate of peripheral naïve T-cell production as described previously (11). Naïve CD4 T cells were identified by staining for CD4 + CD45RA+ lymphocytes, and dividing naïve CD4 T cells were determined using the proliferation marker Ki67. Ki67 is only expressed in proliferating cells in late stage G_I_ then is subsequently rapidly degraded and can, therefore, be used to represent the population of dividing cells.

The equation for thymic export (*θ*) in terms of total naïve cell numbers, naïve cell TREC content and Ki67 expression is given as:

$$\theta \left(t\right)=\frac{y\times N\times \tau }{\Delta \left(c-\tau \right)},$$

where *y* is the fraction of naïve CD4 T cells expressing Ki67, Δ is the duration of Ki67 expression [set to 0.52 (11)], *τ* is the TREC content of the peripheral naïve CD4 T cell population, *c* is a constant representing the average TREC content of thymocytes entering the peripheral naïve population [set to 0.6 (11)], and *N* is the total size of the naïve CD4 T cell pool estimated by multiplying the number of naïve CD4 T cells per microliter of blood by the total volume of blood [0.97 × Log(body weight in kg) + 4.93] then dividing this figure by 0.02 as blood lymphocytes account for approximately 2% of the body’s total lymphocyte population (11).

### Software

Linear and non-linear regression was carried out in the R statistical environment (20). For mixed-effects modeling, we used NON-MEM version 7.3 (21) with the SAEM algorithm for parameter inference, followed by importance sampling to estimate SEs on parameter values and obtain the OFV. The NONMEM control file for the final model is available on request. Perl-speaks-NON-MEM (PsN) was used for covariate analysis (22).

## Results

Four hundred eleven children were enrolled in the CHER trial. Their baseline characteristics by randomization arm are provided in Table S1 in Supplementary Material. Figure 1 shows the numbers of children progressed to each trial stage: starting, interrupting, and restarting therapy according to the protocol.

### CD4-for-Age in Children on Early ART is Stable or Regresses to a Mean Significantly below that of Uninfected Children

**Figure 3:**
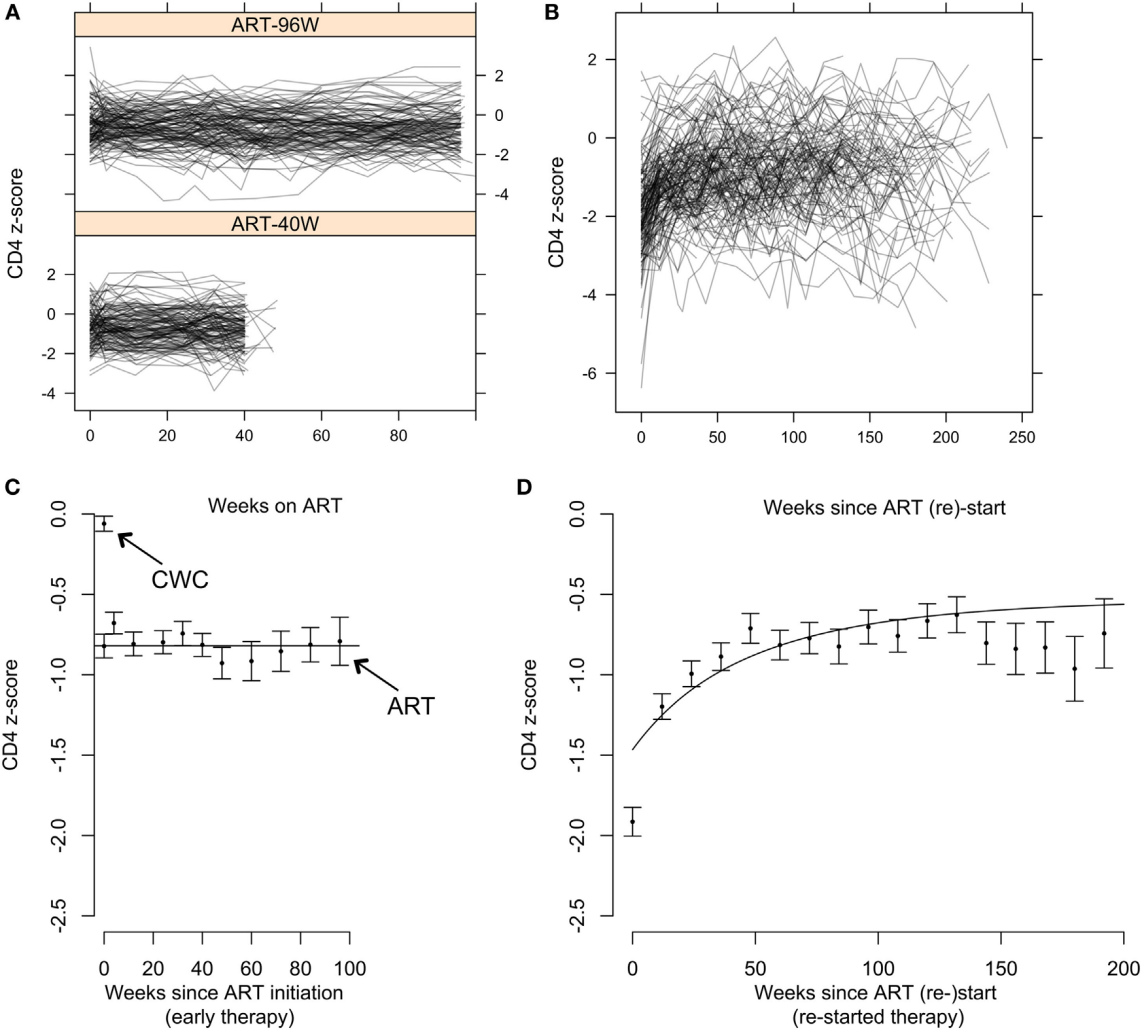
CD4 T-cell dynamics in children on early antiretroviral therapy (ART) and on continuous ART restarted after treatment interruption. **(A)** Individual CD4 *z*-score trajectories for children on early ART for 96 weeks (ART-96W) and 40 weeks (ART-40W). **(B)** Individual CD4 *z*-score trajectories on continuous ART restarted after treatment interruption. **(C)** Means and SEs in the mean (±SEM) of combined ART-96W and ART-40W CD4 *z*-scores for each sampling time on early treatment. The continuous straight line indicates the population-average model fit for the ART-96W and 40 W samples, showing stability of the *z*-scores over time. The *z*-scores in these early-treated children are significantly lower than the mean (±SEM) of the HIV-uninfected Child Wellness Clinic (CWC) cohort. **(D)** Means and SEM of CD4 *z*-scores for each sampling time after ART restart, with the population-average model fit. The discrepancy between the beginning of the curve and the first point is likely to be due to redistribution of memory T-cells in response to ART restart and viral suppression.

Of 284 children starting early ART (141 ART-40W; 143 ART-96W), 10 (4%) had ≤2 CD4 measurements available following ART initiation. CD4 *z*-scores in the remaining 274 are shown in Figure 3A. Of these, 215 (78%) showed no evidence of a trend in CD4 *z*-score with time on ART (*p* > 0.05). There was a downward trend despite ART in 27 (10%) and an increasing trend in 32 (12%). Children with increasing CD4 *z*-score had the lowest CD4 counts and *z*-scores at ART initiation, while children with decreasing scores had the highest (both *p* < 0.0001). Unsurprisingly, children with increasing CD4 *z*-scores also had more advanced disease, while children with decreasing CD4 *z*-scores were more likely to be asymptomatic (*p* = 0.030) (Table S2 in Supplementary Material). These results suggest a regression-to-the-mean phenomenon on early ART, where *z*-scores in children with more advanced disease improve and in children with milder disease decline toward the population average.

Figure 3C shows mean CD4 *z*-scores on early ART. Although there is some fluctuation, it confirms that the CD4 *z*-score was stable during early therapy. Importantly, Figure 3C also shows that even though these children started ART early, aged between 6 and 12 weeks, the stable average CD4 *z*-score was significantly below 0. For comparison, CD4 *z*-scores in >400 HIV-uninfected South African children from the CWC (19) had mean ± SEM −0.06 ± 0.05, significantly higher than the HIV-infected children on early ART. When early ART was initiated, CD4 *z*-scores were comparable to infants randomized to defer treatment (−0.82 ± 0.07 versus −0.64 ± 0.09). By 12 weeks, however, *z*-scores for the ART-deferred cohort had declined to −1.50 ± 0.13 but for the children on ART remained stable at around −0.8 (Figures 3A,C). These results show that early ART over 40 or 96 weeks arrests the decline in CD4 T-cells but does not restore CD4 levels to those in uninfected children.

### CD4 T-Cell Reconstitution in Children Restarting ART after Planned Treatment Interruption

**Figure 4:**
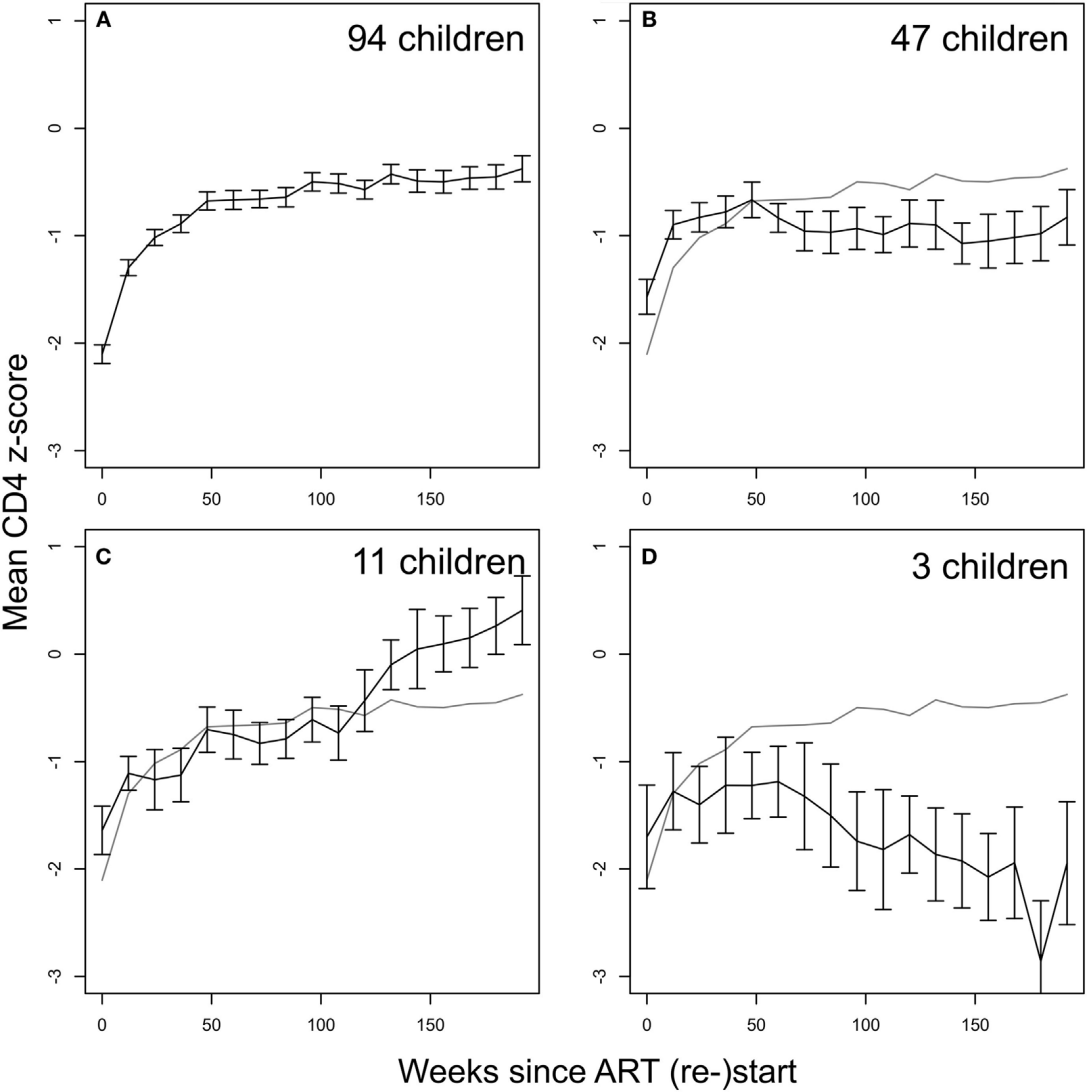
CD4 recovery after restarting continuous antiretroviral therapy (ART) following treatment interruption in ART-40W and ART-96W. **(A)** The majority of children (94, 61%) showed an asymptotic response. **(B)** 47 children had stable average *z*-score after an initial increase, possibly due to CD4 T-cell redistribution. The stable *z*-score at ART restart in these children was significantly lower than that for the asymptotic group. **(C)** 11 children showed continual increase in *z*-scores ending up above the plateau reached in the asymptotic group. **(D)** Decreasing *z*-scores were seen in three children. All error bars give the SE of the mean. The gray line in panels **(B–D)** reproduces the mean *z*-score shown in panel **(A)**.

Children in ART-40W or ART-96W restarted ART after interruption, according to predefined criteria (see Materials and Methods). Figures 3B,D shows CD4 *z*-scores for children restarting ART following planned interruptions. One hundred seventy-two children restarted treatment, of whom seven had no CD4 count available after restarting. The median (IQR) interruption length in the 165 children with at least one CD4 count on continuous therapy was 26 (11–48) weeks. Nineteen of the 165 had interruption length 0 because they were already eligible for ART restart at the interruption date. In 155 of the 165 children who restarted treatment, we identified four distinct response groups similar to those seen in older children (Table S3 in Supplementary Material) (7); CD4 response could not be classified in the remaining 10, who had fewer than three CD4 measurements after restart. In 94 children (61%), CD4 *z*-score increased and then plateaued (“asymptotic reconstitution”; Figure 4A). These children had lower pre-ART-restart CD4 counts (*p* = 0.0005) and *z*-scores (*p* = 0.0002) than the remaining study population but there was no evidence of differences in other characteristics (all *p* > 0.3). In 47 children, CD4 *z*-scores were stable (*p* > 0.05; Figure 4B), but at a lower average level than the asymptotic group. These children had higher CD4 counts (*p* = 0.0027) and *z*-scores (*p* = 0.0011) at ART re-initiation than the asymptotic group. CD4 *z*-scores in 11 children increased to higher levels than those in the other groups, but did not reached a plateau during observation (Figure 4C). These children also had higher CD4 *z*-scores at ART restart than the asymptotic group (*p* = 0.0074), and there was some evidence of higher CD4 counts (*p* = 0.012). CD4 *z*-scores fell with time on ART in only three children (Figure 4D).

### Understanding the Impact of Planned ART Interruption on Long-Term CD4 Levels when Therapy is Restarted

Non-linear mixed-effects modeling was used to identify factors determining the effect of treatment interruption on T-cell reconstitution following ART interruption and better understand its long-term impact. The results are reported in terms of an “average” child for this study: belonging to the largest groups (female, enrolled at PHRU, randomized to ART-40W and restarting ART for clinical reasons) with median enrollment age 8 weeks, restart age 103 weeks, and interruption length 22 weeks. Such an “average” child had stable CD4 *z*-score on early therapy at - 0.79 ± 0.05 (estimate ± SE), corresponding to the 21st centile in healthy children. Following interruption, therapy was restarted in this “average” child at a *z*-score of –1.55 ± 0.16, corresponding to the sixth centile in healthy children. The CD4 *z*-score increased with rate constant 0.016 ± 0.006 weeks^-1^ so that the time to achieve half of the long-term increase (ln2/rate constant) was ~43 weeks. The long-term CD4 *z*-score (asymptote) on continuous restarted therapy was 0.13 ± 0.13 units higher than the stable level on early (pre-interruption) therapy. There was, therefore, no evidence of any decrease in long-term CD4 *z*-score on long-term ART as a result of treatment interruption, relative to the stable *z*-score on early treatment.

The analysis identifies factors influencing CD4 *z*-scores for individual children on early treatment and restarting treatment after planned ART interruption. On early ART, children who were older at ART initiation had lower but stable CD4 *z*-scores (−0.087 ± 0.032 U lower, per week older at enrollment; *p* = 0.016). After treatment interruption, children restarted ART with higher CD4 *z*-scores if they were male (0.252 ± 0.151 U higher; *p* = 0.036), were older when ART was restarted (0.243 ± 0.147 U higher; *p* = 0.032) or, unsurprisingly, if they restarted therapy for clinical (0.331 ± 0.172 U higher) or unspecified (0.666 ± 0.280 U higher) as opposed to immunological reasons (*p* = 0.018). CD4 *z*-score at restart was lower if ART had been interrupted for longer [(−9.98 ± 2.99) × 10^−3^ U lower per week of interruption; *p* = 0.0004]. The increase in CD4 count was exp(0.0140) = 1.01 times faster per week of treatment interruption. Table S4 in Supplementary Material summarizes these results. Notably, there was no evidence that either duration of early therapy (96 versus 40 weeks) or length of interruption predicts CD4 levels on long-term, restarted ART, once other covariates have been taken into account.

Significant correlations were found between children’s CD4 *z*-scores on early therapy, at ART restart after treatment interruption and long-term stable levels (Table S5 in Supplementary Material). This suggests that children have an individual CD4 “set point” established on early therapy, to which they return when therapy is restarted after interruption.

### Naïve CD4 Cells are a Key Determinant of Total CD4 Reconstitution

Age-adjusted naïve and memory cell counts and thymic output were available at three stages: on early ART (66 children), soon after restart (32 children), and in the long term (44 children).

The early stable CD4 *z*-score *prior to treatment interruption* was positively correlated with the naïve CD4-for-age during early treatment and after long-term therapy, and with the memory CD4-for-age on early therapy but *not* the memory CD4-for-age on long-term therapy (Table 1). Thus, early stable CD4 *z*-score level prior to treatment interruption appears to be related to naïve CD4-for-age throughout treatment, but not to predict memory CD4-for-age on long-term therapy.

**Table 1:** Spearman’s correlations (ρ*)* and *p*-values between *z*-scores and naïve and memory cells measured as log ratios to normal age-matched children.

Naïve and memory CD4-for-age ratio to uninfected children		Correlation with CD4 *z*-score obtained from the model		
		On early treatment	At ART restart	In the long term
Early treatment	Naïve	**ρ = 0.61, *p* < 10^−4^**	**ρ = 0.51, *p* < 10^−4^**	**ρ = 0.48, *p* < 10^−4^**
	Memory	**ρ = 0.40, *p* = 0.0009**	ρ = 0.25, *p* = 0.048	ρ = 0.31, *p* = 0.012

Treatment restart	Naive	ρ = 0.43, *p* = 0.014	**ρ = 0.76, *p* < 10^−4^**	**ρ = 0.67, *p* < 10^−4^**
	Memory	ρ = 0.17, *p* = 0.36	**ρ = 0.51, *p* = 0.0030**	**ρ = 0.51, *p* = 0.0033**

Long-term treatment	Naïve	**ρ = 0.69, *p* < 10^−4^**	**ρ = 0.44, *p* = 0.003**	**ρ = 0.83, *p* < 10^−4^**
	Memory	ρ = 0.19, *p* = 0.23	**ρ = 0.45, *p* = 0.0025**	**ρ = 0.50, *p* = 0.0006**

*Bold font is used to highlight *p*-values below 0.01. Naïve and memory cell counts were analyzed using at most one timepoint per participant during early therapy (chosen at random if more than one early-therapy timepoint per child was available); the first available timepoint on continuous therapy, included only if measured at <20 weeks after restarting ART; and the last available timepoint on continuous therapy, included only if measured >100 weeks after restarting ART*.

The CD4 z-score *at ART restart following interruption* was correlated with naïve CD4-for-age before interruption, on restart and in the long term (Table 1). Furthermore, it correlated with memory CD4-for-age at ART restart and long term but not on early therapy. This could be due to changes in memory CD4-for-age related to viral exposure during treatment interruption. This concept is reinforced as the *long-term* CD4 *z*-score was also correlated with naïve CD4-for-age on early therapy, at restart and long term, but with memory CD4-for-age only at restart and long term.

### Thymic Output Drives CD4 Reconstitution

**Figure 5:**
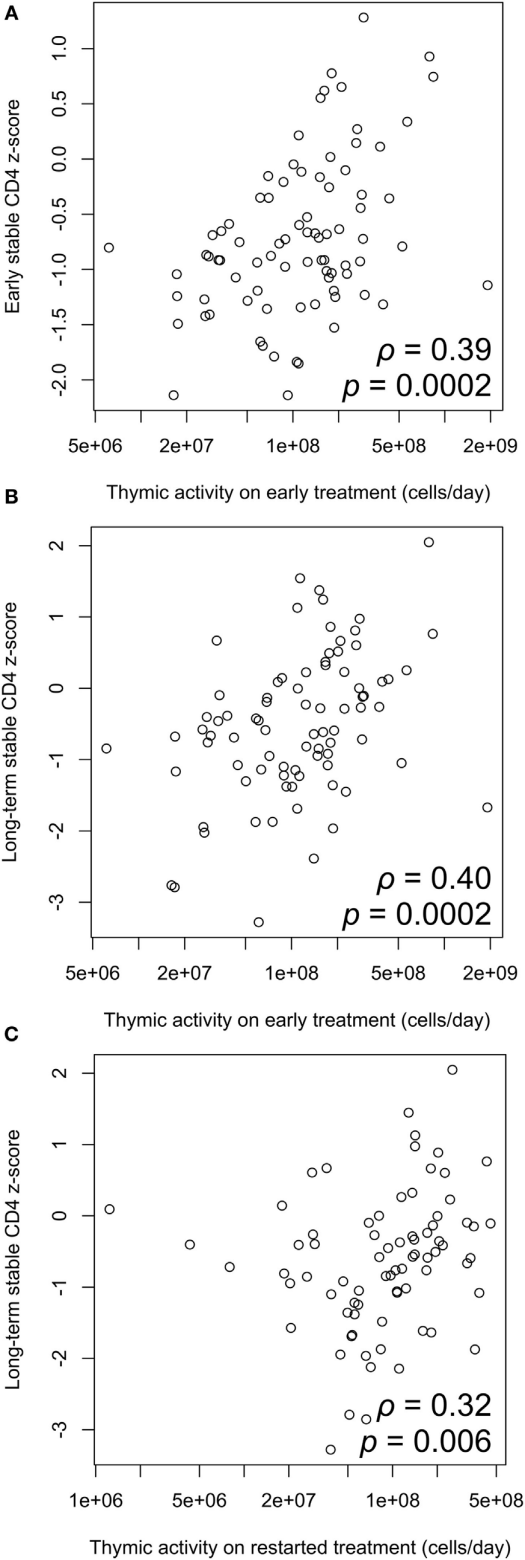
Correlation between thymic output and CD4 *z*-score. **(A)** Thymic output measured on early antiretroviral therapy (ART) is significantly correlated with early stable CD4 *z*-score (ρ = 0.39, *p* = 0.0002). **(B)** Thymic output on early ART is significantly correlated with long-term stable *z*-score after restarting ART (ρ = 0.40, *p* = 0.0002). **(C)** Correlation between thymic output measured on ART restart and long-term stable *z*-score (ρ = 0.32, *p* = 0.006). Correlations determined by two-tailed tests based on the Spearman correlation coefficient ρ.

There is a significant positive correlation between thymic export and *z*-score on early ART, and also the long-term stable *z*-score on ART after treatment interruption (Figures 5A,B). Thymic export measured after treatment restart was also correlated with long-term stable CD4 *z*-score (Figure 5C). These results indicate that the levels of naïve CD4 cells that strongly influence CD4 count before and after interrupting ART arise from thymic output rather than peripheral cell division and that effective T-cell reconstitution depends significantly on the export rate of naïve T-cells from the thymus.

## Discussion

CD4 counts from CHER provide a unique opportunity to explore the dynamics of CD4 reconstitution in HIV-infected infants. During early ART, children’s CD4 *z*-scores were stable but remained significantly below those of uninfected children. Even when ART is started within the first 12 weeks of life it only maintains CD4 levels, rather than reinstating healthy counts.

Most children in the early treatment arms stopped ART at either 40 or 96 weeks. When ART was restarted, according to predefined criteria, a range of CD4 recovery profiles similar to those seen in other studies of older children were observed (7). The largest group experienced an asymptotic rise in CD4 *z*-score followed by a plateau. A smaller group of children saw lesser initial increases but stabilized soon after ART reintroduction; these children’s CD4 *z*-scores had remained relatively high during treatment interruption but remained below the average long-term level in the asymptotic group. In the small group of children in whom CD4 counts increased continuously until the end of the trial, long-term CD4 levels were higher than in the asymptotic group. Only three children had falling CD4 levels following treatment restart.

We showed that long-term CD4-for-age recovery was equivalent to children’s pre-interruption steady state. Although current guidelines do not recommend ART interruption, situations inevitably arise where individual children interrupt because of poor tolerance, family choice, or for logistical reasons. Our analyses suggest that such interruptions in young children who started ART with relatively high CD4s, if planned, monitored and controlled, may not harm long-term CD4 reconstitution. This may provide reassurance in situations where interruptions are unavoidable. The observation is consistent with previous work, suggesting that children starting ART for the first time at young ages have good potential for long-term CD4 recovery (6), for a combination of possible reasons including the preservation of both thymic activity (11, 23) and the machinery of T-cell homeostasis in the lymph nodes (24). It is also consistent with data suggesting planned treatment interruptions in older children do not damage CD4 counts in the long term (8). This remarkable capacity for young children to reconstitute T-cells contrasts with adults in whom treatment interruption is associated with impaired reconstitution (9, 10).

The level of children’s early stable CD4-for-age was predicted by age at first ART initiation. Once started, ART stabilized CD4-for-age for the long term and neither the length of early treatment (96 versus 40 weeks), nor the period of interruption appeared to affect long-term levels. This may be a result of the restricted range of interruption lengths in our study—CD4 trajectories could only be studied in those children who restarted during the 5–6 years of trial data available—but could also suggest that early therapy protects the immune system, especially the thymus, at a crucial developmental phase (12). The correlation between thymic output and CD4 *z*-scores during early treatment and after interruption supports this concept, reinforcing the importance of early diagnosis and treatment to preserve CD4 counts and provide a degree of immunological resilience should treatment be interrupted in the future.

A striking finding was the strong correlation between CD4 levels on early therapy, at ART re-initiation and long term. It seems that children have a CD4 “set point,” established on early ART, to which they return on long-term treatment, even post-interruption. This is consistent with the correlation between CD4 *z*-scores and both thymic output and naïve T-cells and might suggest that the preserved CD4 set-point is dependent on thymic output and naïve CD4 T-cell dynamics. This is in contrast to adults, in whom memory T-cell dynamics play a greater role in reconstitution (25). Despite the high thymic output in young children, most infants treated with early ART achieved maximal CD4 levels significantly below the uninfected children in the CWC cohort. It appears that a CD4 “ceiling” is established *in utero* or in early childhood, which is likely to remain for life. The implications of such a CD4 “ceiling” for children as they age, and particularly in adulthood, are unclear. It does, however, suggest that immunotherapy with, for example, interleukin-7 (26) might be considered to enhance CD4 levels in children.

The main limitation of the study is that we could only examine CD4 trajectories on restarted ART in those children who restarted therapy during the 5–6 years of data available, and not in the 19% of ART-40W and 32% of ART-96W children remaining off ART at the end of the trial. Data from long-term follow-up of these children, or cohort studies, could add to our conclusions in the future by providing outcomes in these children with longer interruptions. Another limitation is the functions used for age-adjustment of total naïve and memory CD4 counts, which are based on reference data from European children. No age-standardization functions for these counts in South African children currently exist, and formulating them would provide a useful clinical and research tool. Finally, CD4 count is only one aspect of the all-round immunological health for which effective HIV management aims. Further monitoring and research into the wider immunological effects of planned treatment interruptions—for example, the possibility of immune activation and associated risks—is important.

This study demonstrates that early identification and treatment of HIV-infected infants is not only important for clinical reasons, but optimizes the CD4 levels achieved with ART in the long term, albeit below levels in uninfected children. Once stabilized, CD4 counts appear resilient, with good potential for recovery following treatment interruption. Our analysis of naïve and memory T-cell subsets and thymic output indicates that the thymus is key to maintaining the naïve pool and determining children’s CD4 levels. This is a major difference between children and adults, and a greater understanding of T-cell dynamics in young children could provide valuable insights into approaches for optimizing immune reconstitution for vertically infected children presenting at older ages (27). The WHO has recently changed its policy, and now recommends universal therapy regardless of CD4 count (28). Long-term studies are required to ascertain how the reduced CD4 ceiling observed for the first time here will influence the health of these children in adulthood.

## Ethics Statement

This study was a secondary analysis of data collected as part of a clinical trial. Research ethics committees in South Africa and the USA approved the trial. Parents or guardians gave written consent for screening and enrollment. Separate ethical approval for the analysis described in this paper was not required.

## Author Contributions

AV, MC, DG, and AB designed the CHER trial. RP managed the trial. KO provided the data used in this analysis. HP carried out the immunology substudy and measurements of thymic activity. JL, RC, and NK designed the analysis, with further contributions from DG, AB, and AW. JL conducted the analysis. JL, RC, and NK wrote the original manuscript. All authors commented on and revised the manuscript.

## Conflict of Interest Statement

JL reports a grant from the Engineering and Physical Sciences Research Council, and travel funding from the British Society for Immunology, during the conduct of the study. AW reports grants from NIH, during the conduct of the study; and fees paid to her institution by Janssen for DSMB membership and Gilead Sciences for lecturing, outside the submitted work. MC reports grants from NIAID, during the conduct of the study; grants from ViiV, other from ViiV (GSK plc), grants from BMS, grants from Gilead, grants from MedImmune, grants from Novartis, grants from VPM, grants from Novavax, personal fees from MSD, outside the submitted work. HP, KO, DG, AB, RP, AV, NK, and RC have nothing to disclose.
