# Supplementary material for: Thymic Output and CD4 T-Cell Reconstitution in HIV-Infected Children on Early and Interrupted Antiretroviral Treatment: Evidence from the Children with HIV Early Antiretroviral Therapy Trial
Source: Front Immunol. 2017 Sep 20;8:1162. doi: 10.3389/fimmu.2017.01162 (PMC5611383; doi:10.3389/fimmu.2017.01162)
Supplement: Supplementary file 1 [file table_1.docx]

**Thymic output and CD4 T-cell reconstitution in HIV-infected children on early and interrupted antiretroviral treatment: evidence from the CHER trial.**

Joanna LEWIS, Helen PAYNE, A Sarah WALKER, Kennedy OTWOMBE, Diana M GIBB, Abdel G BABIKER, Ravindre PANCHIA, Mark F COTTON, Avy VIOLARI, Nigel KLEIN and Robin E CALLARD

**Supplementary Tables**

|  |  | ART-Def (n=125) | ART-40W (n=143) | ART-96W (n=143) | All children (n=411) |
| --- | --- | --- | --- | --- | --- |
| Site | PHRU | 87 (70%) | 103 (72%) | 103 (72%) | 293 (71%) |
|  | KIDCRU | 38 (30%) | 40 (28%) | 40 (28%) | 118 (29%) |
| WHO Stage | N | 105 (84%) | 111 (78%) | 119 (83%) | 335 (82%) |
|  | A | 14 (11%) | 26 (18%) | 14 (10%) | 54 (13%) |
|  | B | 6 (5%) | 6 (4%) | 9 (6%) | 21 (5%) |
|  | C | 0 | 0 | 1 (1%) | 1 (0.2%) |
| Birthweight (kg) |  | 3.00 (2.69, 3.20) | 3.00 (2.71, 3.20) | 2.93 (2.66, 3.25) | 2.98 (2.69, 3.24) |
| Sex (Female) |  | 74 (59%) | 84 (59%) | 64 (45%) | 237 (58%) |
| Age at randomisation (weeks) |  | 7.14 (6.43, 8.86) | 7.57 (6.71, 8.86) | 7.57 (6.57, 8.93) | 7.43 (6.57, 8.86) |
| CD4 count  (cells μL^-1^) |  | 2039 (1585, 2960) | 1978 (1439, 2702) | 2070 (1553, 2762.5) | 2023 (1539, 2791) |
| CD4% |  | 35.6 (28.9, 43.6) | 34.8 (28.9, 40.9) | 34.0 (29.0, 39.3) | 34.9 (28.9, 40.8) |
| CD4 z-score |  | -0.74 (-1.26, 0.09) | -0.79 (-1.40, -0.12) | -0.71 (-1.29, -0.09) | -0.77 (-1.30, -0.06) |

**Table S1: Baseline characteristics of all children enrolled in the CHER trial.** Entries in the table give number (%) for categorical variables, or median (IQR) for continuous variables.

|  |  | Response to early therapy | | | |  |
| --- | --- | --- | --- | --- | --- | --- |
|  |  | <= 2 time points  n=10 | Stable CD4 z-score (p>0.05)  n=215 | Decreasing CD4 z-score (p<0.05)  n=27 | Increasing CD4 z-score (p<0.05)  n=32 | p-value |
| Arm | ART-40W | 5 (50%) | 109 (51%) | 10 (37%) | 17 (53%) | 0.59 |
|  | ART-96W | 5 (50%) | 106 (49%) | 17 (63%) | 15 (47%) | 0.27 |
| Site | PHRU | 9 (90%) | 155 (72%) | 22 (81%) | 20 (63%) |  |
|  | KIDCRU | 1 (10%) | 60 (28%) | 5 (19%) | 12 (38%) |  |
| WHO Stage | N | 10 (100%) | 176 (82%) | 25 (93%) | 19 (59%) | 0.030 |
|  | A | 0 | 29 (13%) | 2 (7%) | 9 (28%) |  |
|  | B | 0 | 10 (5%) | 0 | 3 (9%) |  |
|  | C | 0 | 0 | 0 | 1 (3%) |  |
| Birthweight (kg) |  | 2.81  (2.63, 2.93) | 2.95  (2.65, 3.25) | 3.09  (2.76, 3.26) | 2.79  (3.00, 3.22) | 0.47 |
| Sex (Female) |  | 4 | 124 | 17 | 16 | 0.54 |
| Age (weeks) |  | 6.86  (6.46, 7.93) | 7.57  (6.57, 8.93) | 7.43  (6.93, 8.43) | 7.57  (6.93, 9.18) | 0.53 |
| CD4 count  (cells μL^-1^)^1^ |  | 1987  (1676, 2439) | 2048  (1514, 2736) | 3183  (2219, 4417) | 1631  (1130, 1922) | <0.0001 |
| CD4%^1^ |  | 33.9  (39.5, 46.1) | 35.1  (29.0, 40.6) | 36.3  (29.8, 44.3) | 32.4  (27.5, 37.8) | 0.087 |
| CD4 z-score^1^ |  | -0.78  (-1.14, -0.38) | -0.76  (-1.33, -0.10) | 0.27  (-0.59, 1.13) | -1.15  (-1.81, -0.87) | <0.0001 |

^1^ CD4 count and percentage were unavailable at ART initiation in 15 children.

**Table S2: Pre-ART characteristics of children randomized to early therapy, by CD4 response group on early therapy.** Entries in the table give number (% of children in each response group) for categorical variables, or median (IQR) for continuous variables. P-values are for Fisher’s exact test (categorical variables) or Kruskall-Wallis rank sum test (continuous variables).

|  | |  |  | **Comparisons include all children, randomized to ART-Def, ART-40W and ART-96W** | | | | | | | |  |
| --- | --- | --- | --- | --- | --- | --- | --- | --- | --- | --- | --- | --- |
|  | |  |  | **Comparison of all non-asymptotic responders to asymptotic responders** | | **Comparison of all individual non-asymptotic groups to asymptotic responders** | | | | | |  |
|  | | <= 2 time points | Asymptotic CD4 z-score | Non-asymptotic CD4 z-score | | Stable CD4 z-score (p>0.05) | | Increasing CD4 z-score (p<0.05) | | Decreasing CD4 z-score (p<0.05) | | |
|  | | n=18 | n=156 (63%) | n=93 (37%) | p-value | n=65 (26%) | p-value | n=21 (8%) | p-value | n=7 (3%) | p-value | |
| Arm | ART-Def | 8 | 62 (40%) | 32 | 0.37 | 18 (28%) | 0.054 | 10 (48%) | 0.70 | 4 (57%) | 0.70 | |
|  | ART-40W | 7 | 58 (37%) | 32 |  | 22 (34%) |  | 8 (38%) |  | 2 (29%) |  | |
|  | ART-96W | 3 | 36 (23%) | 29 |  | 25 (38%) |  | 3 (14%) |  | 1 (14%) |  | |
| Site | PHRU | 13 | 97 (62%) | 67 | 0.13 | 47 | 0.17 | 13 (62%) | 1 | 7 (100%) | 0.049 | |
|  | KIDCRU | 5 | 59 (38%) | 26 |  | 18 |  | 8 (38%) |  | 0 |  | |
| WHO  Stage | N | 16 | 120 (77%) | 72 | 0.74 | 49 |  | 17 (81%) | 1 | 6 (86%) | 1 | |
|  | A | 1 | 30 (19%) | 17 |  | 12 | 0.53 | 4 (19%) |  | 1 (14%) |  | |
|  | B | 1 | 6 (4%) | 3 |  | 3 |  | 0 |  | 0 |  | |
|  | C | 0 | 0 | 1 |  | 1 |  | 0 |  | 0 |  | |
| Birthweight (kg) | | 2.98 (2.79, 3.10) | 2.96 (2.66, 3.24) | 3.00 (2.54, 3.25) | 0.87 | 3.00 (2.45, 3.20) | 0.55 | 3.20 (2.74, 3.40) | 0.21 | 2.80 (2.71, 2.97) | 0.36 | |
| Sex Female | | 12 | 92 (36%) | 48 | 0.29 | 34 | 0.37 | 13 (62%) | 1 | 1 (14%) | 0.043 | |
| Male | | 6 | 64 (25%) | 45 |  | 31 |  | 8 (38%) |  | 6 (86%) |  | |
| Age at first ART (weeks) | | 8.8 (6.8, 25.8) | 9.3 (7.4, 22.6) | 9.0 (7.4, 18.7) | 0.62 | 8.7 (7.4, 13.4) | 0.17 | 9.7 (7.4, 24.9) | 0.78 | 11.7 (7.1, 23.5) | 0.93 | |
| Age at ART (re-)start (weeks) | | 54.3 (33.1, 228.4) | 68.1 (32.0, 109.2) | 70.7 (40.0, 126.7) | 0.12 | 95.4 (55.7, 125.7) | 0.012 | 55.7 (36.6, 113.0) | 0.73 | 33.7 (23.5, 63.0) | 0.19 | |
| CD4 count at ART (re-)start (cells μL^-1^)^1^ | | 985 (583, 1287) | 845 (612, 1208) | 1030 (782, 1514) | 0.0057 | 969 (761, 1505) | 0.014 | 1074 (846, 1583) | 0.071 | 967 (768, 1094) | 0.73 | |
| CD4% at ART (re-)start^1^ | | 20.3 (17.0, 29.8) | 20.6 (16.6, 25.9) | 20.3 (16.7, 26.0) | 0.92 | 20.9 (17.0, 25.9) | 0.88 | 18.6 (16.6, 29.0) | 0.75 | 18.6 (16.8 20.8) | 0.63 | |
| CD4 z-score at ART (re-)start^1^ | | -1.79 (-2.78, -1.16) | -2.17 (-2.75, -1.50) | -1.72 (-2.29, -0.91) | 0.0003 | -1.72 (-2.31, -0.83) | 0.0009 | -1.40 (-2.04, -1.05) | 0.019 | -1.94 (-2.44, -1.65) | 0.77 | |

^1^ CD4 count and percentage were unavailable at ART (re-)initiation in 6 children.

**Table S3: Characteristics of children experiencing different responses to ART initiation following delay or interruption.** Entries in the table give number (% of children in each response group) for categorical variables, or median (IQR) for continuous variables. P-values are for Fisher’s exact test (categorical variables) or Kruskall-Wallis rank sum test (continuous variables). Note that the table compares children enrolled in all three arms of the trial (ART-Def, ART-40W and ART-96W), whereas comparisons reported in the text compared only children randomized to early treatment (ART-40W and ART-96W).

| Model Parameter | Covariate |  | Estimate | Standard error | p-value |
| --- | --- | --- | --- | --- | --- |
| Early stable level | Typical value |  | -0.793 | 0.0638 |  |
|  | Age at enrollment (weeks) |  | -0.087 | 0.032 | 0.016 |
| Re-start level | Typical value |  | -1.55 | 0.16 |  |
|  | Sex | Male | 0.252 | 0.151 | 0.036 |
|  | Reason for restart | Clinical | 0.331 | 0.172 | 0.018 |
|  |  | Unspecified | 0.666 | 0.280 |  |
|  | Age at ART restart (years) |  | 0.243 | 0.147 | 0.032 |
|  | Length of interruption (weeks) |  | -9.98 × 10^-3^ | 2.99 × 10^-3^ | 0.0004 |
| Difference in stable levels | Typical value |  | 0.125 | 0.137 |  |
|  | Site | KIDCRU | 0.427 | 0.214 | 0.011 |
| Rate of z-score increase | Typical value |  | 0.0160 | 0.00551 |  |
|  | Length of interruption (weeks) |  | 0.0140 | 0.00844 | 0.020 |

**Table S4: Covariate models of the parameters in the mixed-effect model.** Covariates identified by our model-building process as predictive of CD4 parameters describing CD4 z-score trajectory are included. P-values are reported for an approximation to the likelihood ratio test, comparing the improvement in objective function value (-2 *log-likelihood) to a chi-squared distribution. “Typical value” parameters give the value estimated for an “average” child as described in the main text (in the largest groups for categorical variables, and with median values of other characteristics). We also provide the estimated and standard error effect sizes of each covariate identified as significant, on each parameter.

|  | Early stable CD4 z-score | Z-score at ART re-initiation | Long-term stable z-score | Rate of z-score increase (weeks^-1^) |
| --- | --- | --- | --- | --- |
| Early stable CD4 z-score | 0.47 ± 0.06 | 0.59 ± 0.10 | 0.65 ± 0.08 | (-4.12 ± 349) × 10^-3^ |
| Z-score at ART re-initiation | 0.36 ± 0.07 | 0.82 ± 0.16 | 0.53 ± 0.07 | -0.44 ± 0.20 |
| Long-term stable z-score | 0.46 ± 0.09 | 0.50 ± 0.09 | 1.07 ± 0.22 | -0.20 ± 0.17 |
| Rate of z-score increase (weeks^-1^) | (-2.62 ± 222) × 10^-3^ | -0.37 ±0.22 | -0.20 ± 0.19 | 0.87 ± 0.42 |

**Table S5: Correlations and covariances between random effects (child-specific) parameter values in our model.** Values on and below the diagonal (white cells) are variances and covariances. Values above the diagonal give correlations. All values are reported as (value ± standard error).
